# Green tea catechin, epigallocatechin-3-gallate, attenuates the cell viability of human non-small-cell lung cancer A549 cells via reducing Bcl-xL expression

**DOI:** 10.3892/etm.2014.1719

**Published:** 2014-05-19

**Authors:** JUN-ICHIRO SONODA, RYUJI IKEDA, YASUTAKA BABA, KEIKO NARUMI, AKIO KAWACHI, ERISA TOMISHIGE, KAZUYA NISHIHARA, YASUO TAKEDA, KATSUSHI YAMADA, KEIZO SATO, TOSHIRO MOTOYA

**Affiliations:** 1 First Department of Clinical Pharmacy, School of Pharmaceutical Sciences, Kyushu University of Health & Welfare, Nobeoka, Miyazaki 882-8508, Japan; 2 Department of Clinical Pharmacy and Pharmacology, Graduate School of Medical and Dental Sciences, Kagoshima University, Sakuragaoka, Kagoshima 890-8520, Japan; 3 Department of Radiology, Graduate School of Medical and Dental Sciences, Kagoshima University, Sakuragaoka, Kagoshima 890-8520, Japan; 4 Department of Clinical Pharmacology, Faculty of Pharmaceutical Science, Nagasaki International University, Sasebo, Nagasaki 859-3298, Japan; 5 Department of Clinical Biochemistry, School of Pharmaceutical Sciences, Kyushu University of Health & Welfare, Nobeoka, Miyazaki 882-8508, Japan

**Keywords:** epigallocatechin-3-gallate, A549 cells, B-cell lymphoma-extra large, apoptosis

## Abstract

Clinical and epidemiological studies have indicated that the consumption of green tea has a number of beneficial effects on health. Epigallocatechin-3-gallate (EGCg), the major polyphenolic compound present in green tea, has received much attention as an active ingredient. Among the numerous promising profiles of EGCg, the present study focused on the anticancer effects. Apoptosis induced by EGCg and subsequent cell growth suppression have been demonstrated in a number of cell culture studies. However, the underlying mechanism of apoptotic cell death remains unclear. Thus, the aim of the present study was to identify the major molecule that mediates proapoptotic cell death by EGCg. The effect of EGCg on cell proliferation and the induction of mRNA that modulates apoptotic cell death was evaluated in the A549 human non-small-cell lung cancer cell line. In addition, morphological changes were assessed by microscopy in A549 cells that had been treated with 100 μM EGCg for 24 h. The MTT assay revealed that cell proliferation was significantly reduced by EGCg in a dose-dependent manner (3–100 μM). The mRNA expression level of B-cell lymphoma-extra large (Bcl-xL) was decreased in A549 cells following 24 h incubation with 100 μM EGCg. Therefore, the results indicated that the inhibition of cell proliferation by EGCg may be achieved via suppressing the expression of the cell death-inhibiting gene, Bcl-xL.

## Introduction

**Figure 1:**
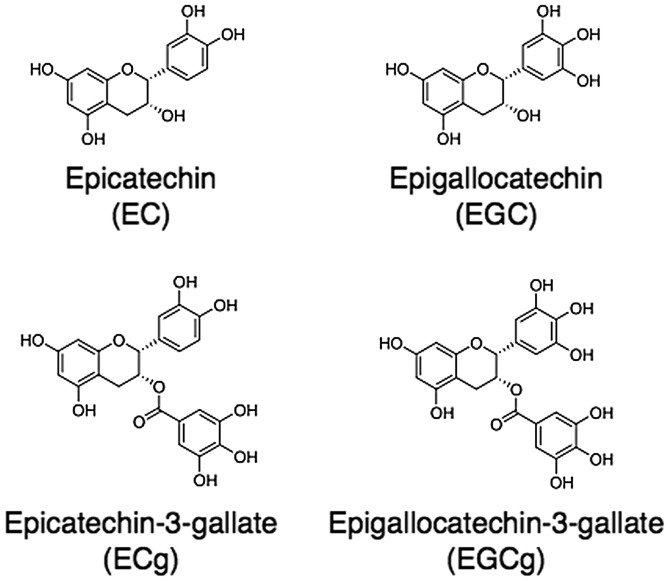
Chemical structures of green tea catechins.

Green tea (*Camellia sinensis*) is one of the most popular beverages worldwide, and contains a large amount of flavonoids, predominantly catechins, including epicatechin, its hydroxyl derivative epigallocatechin, and their gallic acid esters, epicatechin-3-gallate and epigallocatechin-3-gallate (EGCg; Fig. 1). Among these catechins, EGCg is an abundant constituent of green tea (leaf) and has been shown to exhibit antioxidative, anticarcinogenic and anticancer effects *in vitro*. The polyphenolic structure of these compounds exerts antioxidative effects by trapping reactive oxygen species (ROS). It was previously demonstrated that daily intake of green tea reduced oxidative stress *in vivo* (1). In addition, against a background of increasing public health concerns, it has been hypothesized that green tea consumption has beneficial effects against various pathological conditions, including cardiovascular disease, diabetes and cancer.

Green tea catechins, in particular, have attracted attention as cancer-preventive agents due to their low toxicity and ready availability to the general population, as well as exerting preventive effects against cancers in humans (2–5). A prospective cohort study on a Japanese population demonstrated that green tea has a strong potency in preventing cancers in a variety of organs (6). Additional epidemiological or clinical studies revealed that green tea consumption is inversely associated with the progression of prostate cancer, the risk of hematological malignancies and the risk of breast cancer recurrence, among others (7–9). In cells cultured for *in vitro* experiments on green tea catechins, growth inhibition and apoptosis induction have been observed in a variety of cell lines (10,11). Previously, using two cell lines, peripheral blood T lymphocytes of adult T-cell leukemia patients and human T-cell leukemia virus type 1 (HTLV-1)-infected T-cell line, it was demonstrated that EGCg inhibited cell growth concomitant with the induction of apoptosis, and was responsible for suppressing the expression of HTLV-1 pX mRNA, which encodes the oncoprotein, Tax (12). Tax protein plays an important role in HTLV-1-infected T-cell leukemogenesis by mediating interactions with transcription factors, including nuclear factor (NF)-κB. In the CTLL-2 Tax-expressing mouse T-cell line, constitutive expression of B-cell lymphoma-extra large (Bcl-xL) via the NF-κB pathway has been shown to contribute to the inhibition of apoptosis (13).

Several cell culture studies have focused on one of the hallmarks of the decrease in cell growth by green tea catechins, namely, the suppression of NF-κB activation and the subsequent induction of apoptosis. However, *in vivo* evidence remains limited and no definitive conclusions have yet been drawn. Ahmad *et al* revealed that EGCg reversed the degradation of IκBα protein, which specifically inhibits NF-κB activation, and subsequently downregulated cell cycle deregulation and the induction of apoptosis in A431 human epidermoid carcinoma cells (14). An *in vivo* interventional study revealed that intake of green tea extract capsules diminished the HTLV-1 provirus load in peripheral blood lymphocytes of asymptomatic HTLV-1 carriers. Therefore, it was hypothesized that the decrease in HTLV-1 provirus load was caused by EGCg stabilizing IκB and abrogating NF-κB activation in HTLV-1 carrier lymphocytes following the intake of capsules (15).

An increase in the level of nuclear translocation or constitutive activation of NF-κB has been attributed to the induction of prosurvival gene products, including Bcl-2 and Bcl-xL (16,17). Bcl-xL, a member of the Bcl-2 family, inhibits apoptosis by blocking the release of cytochrome *c* from the mitochondria. A decrease in Bcl-xL gene expression may lead to the promotion of cell death. However, the events downstream of NF-κB inactivation by catechins are not clear.

Among green tea catechins, EGCg has been shown to exhibit optimal anticancer activity, which is associated with the number of -OH groups. Therefore, in the present study, EGCg was adopted as a well-characterized model catechin. The aim of the present study was to identify the major molecule that mediates proapoptotic cell death by EGCg. To achieve this objective, the A549 human non-small-cell lung cancer cell line was used and the effect of EGCg on cell proliferation and the induction of mRNA that modulates apoptotic cell death was evaluated.

## Materials and methods

### Chemicals and reagents

EGCg was purchased from Funakoshi Co., Ltd. (Tokyo, Japan). RPMI-1640 medium and 100X Antibiotic-Antimycotic were obtained from Invitrogen Life Technologies (Carlsbad, CA, USA), while fetal bovine serum (FBS) was purchased from Thermo Scientific Fisher (Waltham, MA, USA). Reverse transcription polymerase chain reaction (RT-PCR) was performed with the SuperScript One-Step RT-PCR with Platinum *Taq* kit (Invitrogen Life Technologies) and total RNA was extracted using TRIzol reagent (Invitrogen Life Technologies). MTT assay kit was obtained from Roche Diagnostics (Indianapolis, IN, USA). EGCg was dissolved in phosphate-buffered saline (PBS) as a 2 mM stock solution and then stored at −30°C.

### Cell lines and cell culture

A human non-small-cell lung cancer cell line, A549, was provided by Professor Akiyama from the Department of Molecular Oncology at the Graduate School of Medical and Dental Sciences (Kagoshima University, Kagoshima, Japan). A549 cells were grown in RPMI-1640 medium supplemented with 10% FBS and Antibiotics-Antimycotics in a 5% CO_2_ humidified atmosphere at 37°C.

### Determination of cell survival using the MTT assay

Chemosensitivity was measured *in vitro* using the MTT colorimetric assay, which was performed in 96-well plates (18). To determine the effect of EGCg, A549 cells (2.5×10^3^) in 90 μl culture medium were inoculated into each well. Following 24 h incubation, 10-μl samples of various concentrations of EGCg and the vehicle were added and the plate was incubated for 72 h. Next, 0.5 mg/ml MTT (final concentration) was added to each well and the plate was incubated for a further 4 h at 37°C. The resulting formazan was dissolved in 100 μl solubilization solution (10% SDS in 0.01 M HCl) and the plate was re-incubated overnight at 37°C. The optical density (OD) at 550 nm was determined using an ARVO SX model 1420 Multilabel Counter (PerkinElmer, Waltham, MA, USA). The control wells were set as zero absorbance. The percentage of cell survival was calculated using the background-corrected absorbance as follows: Cell survival (%) = (OD_experiment_/OD_control_) × 100. The data represent the mean and standard deviation from triplicate determination.

### RT-PCR

Total cellular RNA was extracted using TRIzol reagent, according to the manufacturer’s instructions. RT-PCR was performed with the SuperScript One-Step RT-PCR system and gene-specific primers, according to the manufacturer’s instructions. The reaction mixture contained 500 ng total RNA, 0.2 mM dNTPs, 0.2 μM each primer and the enzyme mixture, including SuperScript II RT, Platinum *Taq* DNA polymerase and 1X buffer with 1.2 mM MgSO_4_. The mixture was maintained at 50°C for 20 min, 94°C for 2 min and then PCR was performed as follows: 30 cycles at 94°C for 15 sec, 55°C for 30 sec and 70°C for 30 sec. The primers for RT-PCR were designed on the basis of the human sequences in GenBank. These sequences used the following primers: Bcl-xL primer forward, 5′-CGGTGAATGGAGCCACTGACCA-3′ and reverse, 5′-GCCATCCAAGCTGCGATCCGAC-3′; GAPDH forward, 5′-AGAACATCATCCCTGCCTCTACTGG-3′ and reverse, 5′-AAAGGTGGAGGAGTGGGTGTCGCTG-3′.

### Statistical analysis

Data from MTT assay were presented as the mean ± standard deviation of triplicate determinations. Statistical difference was analyzed using a one-way analysis of variance (ANOVA) followed by Dunett’s test. SPSS software (SPSS Inc., Chicago, IL, USA) was used and P<0.05 was considered to indicate a statistically significant result.

## Results

### Effect of EGCg on the proliferation of A549 cells

**Figure 2:**
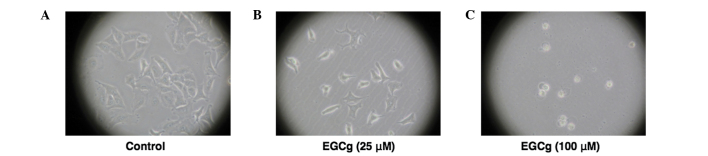
Effect of EGCg on the morphology of A549 cells. Representative morphology of A549 cells was microscopically observed in (A) control and following co-culture with (B) 25 μM and (C) 100 μM EGCg. EGCg, epigallocatechin-3-gallate.

**Figure 3:**
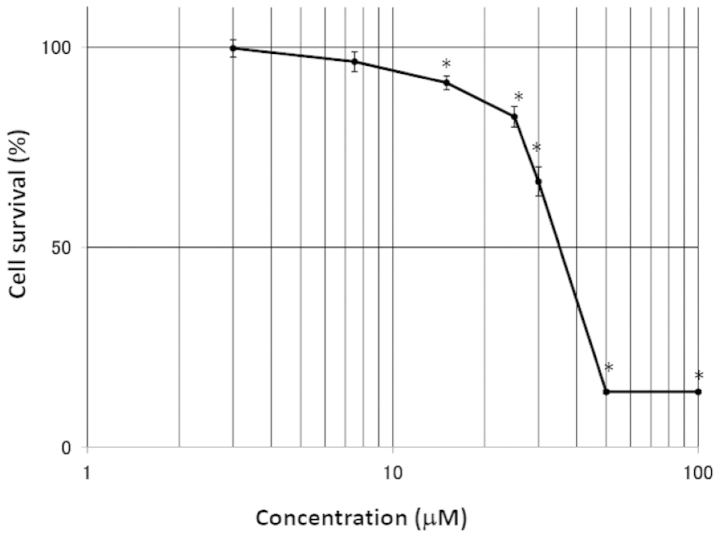
Effect of EGCg on A549 cell survival in the absence or presence of EGCg (3–100 μM), as determined by an MTT assay. Points represent the mean of triplicate determination and the bars show the standard deviation. EGCg, epigallocatechin-3-gallate. *Significant reduction compared with control (0 mM EGCg).

Morphological changes in A549 cells were shown to be dependent on the EGCg concentration. Cells exhibited a shape representative of A549 cells in the control (24 h incubation), whereas the cells lost their adhesion ability when treated with 25 μM EGCg. When treated with 100 μM ECGg, the cells were observed to float in the medium, exhibiting cell death (Fig. 2). The MTT assay was performed at 48 h after treatment with EGCg in the A549 cells. As shown in Fig. 3, the survival rate in the A549 cells was significantly suppressed by treatment with EGCg. The cell viability rate was markedly reduced at EGCg concentrations >25 μM, reaching a plateau at 50 μM. The IC_50_ (50% inhibition of cell growth) for EGCg in the A549 cells was 36.0 μM.

### Effect of EGCg on the mRNA expression of Bcl-xL in A549 cells

**Figure 4:**
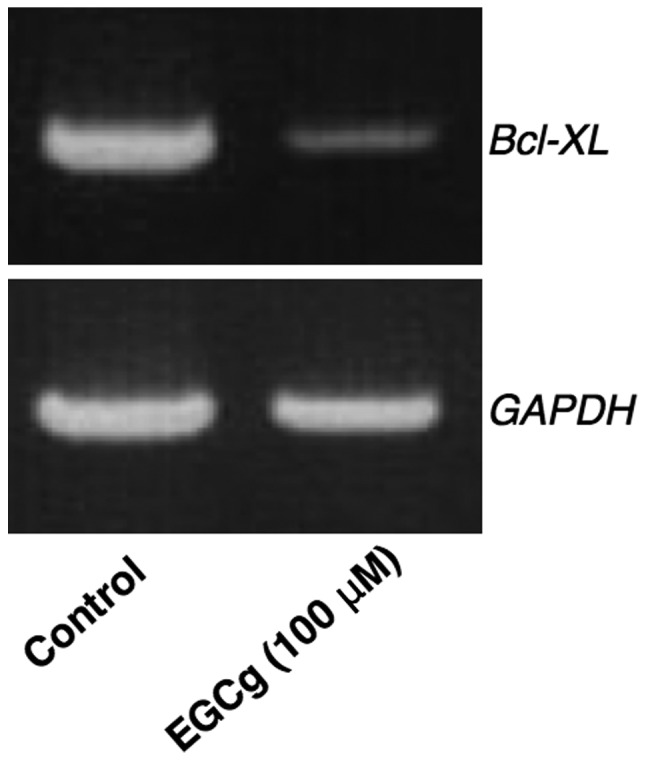
Effect of EGCg on the expression of Bcl-xL in A549 cells, as determined by RT-PCR analysis. Representative image showing the Bcl-xL and GAPDH mRNA expression levels in the absence or presence of 100 μM EGCg. EGCg, epigallocatechin-3-gallate; Bcl-xL, B-cell lymphoma-extra large; RT-PCR, reverse transcription polymerase chain reaction.

Intracellular Bcl-xL expression was analyzed since this protein strongly inhibits apoptosis. If cytosolic Bcl-xL mRNA expression was suppressed by EGCg, the target cells should be induced to undergo apoptosis. The effect of EGCg on the expression of Bcl-xL in A549 cells is shown in Fig. 4. EGCg (100 μM) was shown to suppress the mRNA expression of Bcl-xL in A549 cells at 24 h following administration.

## Discussion

In the present study, EGCg was demonstrated to markedly inhibit cell proliferation at concentrations between 25 and 100 μM (Fig. 3), and decrease Bcl-xL mRNA expression under the same conditions at 100 μM (Fig. 4) in A549 cells. EGCg has been reported to inhibit the activation of NF-κB (14), and the activation of NF-κB leads to the inhibition of apoptosis. NF-κB is a heterodimer consisting of two proteins, p65 and p50. In unstimulated cells, NF-κB is located in the cytoplasm and is bound to IκBα and IκBb, which prevents the molecule from entering the nucleus. External stimuli modulate signal transduction pathways leading to IκB phosphorylation, causing its rapid degradation by proteasomes. The release of NF-κB from IκB results in translocation to the nucleus, where NF-κB binds to a specific sequence in the promoter regions of target genes of antiapoptotic proteins, including Bcl-xL. Therefore, the results of the present study indicate that EGCg reduces the expression of the death-inhibiting gene, Bcl-xL, consequently inducing apoptosis in A549 cells.

Although green tea catechins have been shown to reduce the risk of cardiovascular disease and certain types of cancer, as well as promote physiological functions, including body weight control and antihypertensive, antibacterial, antiviral and neuroprotective effects (19), the present study focused on the anticancer effect of EGCg. On the basis of recent observations, green tea catechins were assumed to exhibit three beneficial properties against cancer.

Firstly, green tea catechins, predominantly EGCg, have potent antioxidant activity and may reduce adverse events associated with pro-oxidant anticancer agents. Generally, anthracyclins and a platinum agent (cisplatin) are considered to release ROS and cause unique side effects, namely, cardiac toxicity and renal dysfunction, respectively. Green tea catechins have been shown to protect against normal cell damage from ROS. Previously, it was demonstrated that daily intake of green tea tablets containing 474 mg catechins significantly reduced the oxidative stress induced by hepatic arterial infusion of cisplatin and 5-fluorouracil in patients with metastatic liver cancer or hepatocellular carcinoma (1). It has also been indicated that administration of EGCg together with pro-oxidant anticancer agents is useful in minimizing adverse effects (20,21). In Japan, cisplatin combination regimens have been considered as standard chemotherapy for non-small cell lung cancer (NSCLC) (22). We hypothesized that conventional chemotherapy combined with green tea catechins may be useful for enhancing their anticancer effectiveness and reducing their adverse drug reactions. Therefore the A549 cell line, which is derived from NSCLC, was adopted in this study assuming lung cancer therapy.

Secondly, EGCg has shown the reverse property against multidrug resistance (MDR). Upon exposure to one chemotherapeutic agent in a clinical context, cancer cells may acquire resistance to chemotherapy. Overexpression of efflux transporters, including P-glycoprotein (P-gp), multidrug-resistance-associated protein 1 and breast cancer resistance protein, has been shown to be a major cause of MDR. Green tea catechins are one type of candidate agent for an effective MDR modulator since they exhibit few side effects and are consumed routinely by a number of people, as a therapeutic aid. A previous study demonstrated that EGCg reversed a doxorubicin-resistant model of hepatocellular carcinoma by inhibiting P-gp pump function (23).

Finally, the most crucial feature is that green tea catechins themselves possess anticancer activity. The present study demonstrated that EGCg significantly reduced A549 cell proliferation at a concentration of 100 μM. Numerous studies on a wide variety of histological types of cancer, including prostate, breast, colorectal, esophageal, stomach and pancreatic cancer, have also documented the anticancer effects of green tea catechins in experimental and clinical studies, as well as in population-based studies (24–32).

Numerous cell-culture studies have revealed that green tea catechins, particularly EGCg, exert growth inhibition and apoptosis induction effects. Apoptotic cell death is mediated by regulator proteins, including Fas ligand, tumor necrosis factor-α, CD95, NF-κB, apoptotic protease activating factor 1 (Apaf-1), caspases and the Bcl-2 family (Bcl-2, Bcl-xL, Bax and Bad). In human cancer cell lines, it has been demonstrated that [^3^H]EGCg or fluorescein isothiocyanate-conjugated EGCg is incorporated into the cytosol and the nucleus in a time-dependent manner (33,34). The structure of EGCg was shown to be preserved in the cytosol following identification with a high performance liquid chromatography-electrochemical detector with a reversed-phase column, and the retention time of cytosolic EGCg matched that of standard EGCg (35). Thus, antiapoptotic function evoked by EGCg may be localized in the cytosol, and EGCg may interact with intracellular proteins, including the IκB/NF-κB complex, caspases, Apaf-1 and the Bcl-2 family (Bcl-xL, Bcl-2, Bax and Bad). Certain studies have demonstrated that EGCg inactivates NF-κB, which consequently induces apoptosis (14,36). The present study attempted to identify a common molecule that is closely associated with apoptosis induction by EGCg, and we hypothesize that the decrease in Bcl-xL expression levels is accompanied with the downstream inactivation of NF-κB. The expression levels of antiapototic protein, Bcl-xL, have been shown to be regulated by the NF-κB transcription factor in a wide spectrum of cells (10,13,37). In the present study, Bcl-xL mRNA expression levels were shown to be reduced following treatment with EGCg in A549 cells.

In conclusion, the results of the present study demonstrate that the inhibition of cell proliferation by EGCg may occur via the suppression of cell death-inhibiting gene expression. Bcl-xL mRNA expression levels decreased following EGCg administration in non-small-cell lung cancer A549 cells. The observations indicate that green tea may be useful as an antitumor agent to enhance the efficacy of cancer therapy. Although the balance between the expression levels of death-inhibiting genes (Bcl-xL and Bcl-2) and death-promoting genes (Bax and Bad) is critically important in the regulation of apoptosis, whether EGCg affects Bcl-2, Bax or Bad gene expression is unclear at present. Thus, further studies investigating whether EGCg regulates the gene expression of Bcl-2 family members other than Bcl-xL are required.
